# Communicative Coping and Friendship Among Persons Living With Dementia: Findings From Long-Term Care

**DOI:** 10.1093/geroni/igab046.1386

**Published:** 2021-12-17

**Authors:** Pamela Saunders

**Affiliations:** Georgetown University, WASHINGTON, District of Columbia, United States

## Abstract

The study of identity is central to many disciplines, however there is a special link that connects language and discourse to identities. The way people speak reveals a lot about who they are. Through discourse and communication individuals convey and negotiate their sense of self (de Fina, 2020). Regardless of cognitive status, persons living with dementia (PLWD) use language to construct for themselves a social identity of being included in friendship networks (de Medeiros et al., 2011). This paper uses data from the Friendship Study to examine the use of such communicative coping behavior (CCB) for friendship formation. Ethnographic observations of PLWD were conducted in a Long-Term Care residential setting. Sociolinguistic discourse analysis of verbatim transcripts with reference to the CCB Checklist (Saunders et al., 2016) reveal evidence of CCB use. Results suggest that different types of CCBs were used to construct identity and negotiate friendship challenges in different contexts.

